# Medical Societies

**Published:** 1879-11-20

**Authors:** 


### *MEDICAL SOCIETIES.*

We have repeatedly urged our medical brethren to form medical societies. They serve the double purpose of mutual improvement and of cultivating a degrée of social feelng and brotherhood, which otherwise will not be likely to exist in a community where two or more physicians are thrown in competition. It is a lamentable fact that instead of kindly and social relations between medical brethren, the opposite condition of things too often obtains.

Nothing so quickly dispels this unfriendliness as a medical society at which physicians are thrown together and talk over their cases and their troubles.

We would cheerfully encourage the formation of societies for the reasons mentioned. It is important also as a means of medical progress The interchange of ideas and methods of practice is certainly promotive of mutual improvement in the practice, and tends by the development of new facts and the diffusion of useful experiences to add to the general progress of the profession everywhere. We will say to any who will organize a society in their midst that we will club the JOURNAL to the members of the same at greatly reduced rates.

